# Can clean energy and technology address environmental sustainability in G7 under the pre-set of human development?

**DOI:** 10.1007/s11356-024-32011-y

**Published:** 2024-01-24

**Authors:** Shaibu Ali, Khatib Ahmad Khan, Bright Akwasi Gyamfi, Elvis Kwame Ofori, Derrick Tetteh, Zilola Shamansurova

**Affiliations:** 1 School of Finance and Economics, Jiangsu University, Xuefu Road, Zhenjiang, Jiangsu China; 2 School of Commerce and Management Studies, SunRise University, Alwar, Rajasthan India; 3 School of Business, Xi’an International University, Xi’an, China; 4 School of Management, Sir Pandampat Singhanian University, Udaipur, Rajasthan India; 5 Plants & Agribioscience, School of Biological & Chemical Sciences, Ryan Institute, University of Galway, Galway, Ireland; 6 School of Management Engineering, Zhengzhou University, Henan Province, Zhengzhou, People’s Republic of China; 7 School of Business, University of Cape Coast, Cape Coast, Ghana; 8 Department of Finance, Tashkent State University of Economics, Tashkent, Uzbekistan

**Keywords:** COP 27, Clean energy, Sustainable development, Technology innovation, Human development, G7

## Abstract

Climate change presents challenges for both industrialized and developing nations, primarily due to insufficient pollution control. Increased fossil fuel usage escalates pollution levels, emphasizing the need to integrate more renewable energy into the energy mix, particularly to reduce carbon emissions. Consequently, public investment in renewable energy becomes pivotal to enhance the necessary technology for green energy production. Human development and technological progress play a crucial role in advancing green energy and ensuring environmental sustainability. This study addresses whether clean energy and technology can foster ecological sustainability in the G7 while considering human development. Findings emphasize the significance of public investments in renewable energy projects, technical innovation, and human development. Such investments are essential for augmenting renewable energy shares and lowering carbon emissions in the long run. The study proposes relevant policies to help G7 nations achieve United Nations Sustainable Development Goals related to green energy transition (SDG-7), environmental sustainability (SDG-13), and innovation (SDG-9). In essence, prioritizing renewable energy investment and innovation is imperative for sustainable development.

## Introduction

In the early 1990s, the environmental Kuznets curve (EKC) emerged as the primary theory to explain the relationship between economic growth and environmental deterioration (Grossman and Krueger 1991). This underlining situation prompted the call for sustainability in energy-environmental literature. Even with efforts to make clean, modern energy more widely available, the majority of people on the planet still cook with conventional, environmentally harmful fuels and appliances. Roughly 2.8 million individuals, according to the Energy Sector Management Assistance Program (ESMAP 2020) study, continue to use conventional, ecologically harmful fuels and cooking methods. Serious issues with the ecology, economy, and health arise from cooking with excessive dependence on traditional, dirty fuels and technology. For example, research available indicates that cooking using old polluting fuels and technology has caused over four million indoor pollution-related fatalities annually and has further cost the world economy over 2.4 trillion dollars. What is more, according to IRENA (2019), the costs associated with using unclean cooking fuels and technology on women’s health are predicted to be $1.4 trillion and $0.8 trillion, respectively. The considerable financial, healthcare, and environmental costs of using conventional polluting fuels and technologies need governments to emphasize policies and methods for hastening the transition to clean fuels.

On the industrial side, firms are also resistant to transition from fossil input for production adding to the existing problem of climate change. Given these challenges, cleaner energy alternatives have caught the attention of all nations as a crucial energy source to curtail carbon dioxide emissions and halt the contamination of the environment (Yang et al. 2022a, b, c; Yang et al. 2022a, b, c). Additionally, the International Renewable Energy Agency said in 2017 that the shift to a future powered by renewable energy is now widely acknowledged as a crucial component of the fight against climate change (IRENA 2017). As a result, several nations have made significant investments in renewable energy. However, the market penetration of renewable energy sources is currently insufficient to effectively prevent environmental harm, despite the substantial efforts made by the various governments (Aghahosseini et al. 2023). So, in order to move forward with reaching the SDGs, hurdles to the adoption of renewable energy policies must be adequately addressed (Ahmed et al. 2022a, b; Apergis et al. 2018).

Despite the widespread assumption that most industrialized nations (G7) are technologically competent (Ahmad and Brosio 2022; Herman and Xiang 2022), these nations have not yet fully adopted the necessary technology to decrease their reliance on conventional fossil fuels and transition to contemporary energy (Quito et al. 2023; Shrestha et al. 2022). Resultantly, fossil fuels account for a significant portion of the national energy mix (Ahmed et al. 2022a, b; Quadrelli and Peterson 2007; Zeng et al. 2021). Hence, a plausible hypothesis emerges: Industrialized nations possess the potential to mitigate the ecological impact stemming from fossil fuel consumption by increasing investment in technological innovation and research and development (R&D), allowing long-term discovery of cutting-edge technical know-how regarding the shift toward sustainable energy alternatives (Bekun 2022; Ma et al. 2022). Therefore, the reprise indicates that the environmental effects of economic development, energy usage (clean), and technological advancement might be unclear.

Despite a wealth of literature, environmental problems continue to grow, necessitating a solution beyond the conventional viewpoint-promoting new literature using human development capital as an intervention to augment the existing solution (Amuakwa-Mensah and Näsström 2022; Budhwar et al. 2017; Dai et al. 2023; Nchofoung et al. 2022; Zallé, 2019). Scholars have proven that transitioning to sustainable development is difficult without the participation of knowledgeable people and decision-makers (Hao 2022). As a result, human capital development, which is linked to excellent education (SDG4), is a critical component of sustainable development (Festus Victor Bekun et al. 2023a, b; Pradhan et al. 2017). Thus, human capital with an educational foundation may impact ecological quality (Chen et al. 2022). High human capital may minimize environmental pollution by using more cutting-edge technologies. With increased knowledge, people may be more inclined to pay carbon taxes and choose ecologically friendly foods and renewable energy sources. Existing empirical works lay claim to this assertion (Chen et al. 2022). According to Alola et al. (2021), human capital in high-income states reduces ecological footprint but increases in low-income groups and nations with huge populations. Human development can indeed augment environmental woes within industrialized countries. Following this, we can posit that increased human capital may help people better comprehend ecological and energy security challenges, which will motivate them to work more productively in present circumstances (Alola et al. 2023a, b; Li and Ullah 2022). Additionally, it allows less energy to be utilized per unit output by lowering energy intensity.

The current study covers a research gap in the prior literature by including access to clean energy and technology as a supplement source of clean energy instead of renewable energy that provides for certain biomass components as predictors of ecological footprint in G7. We concentrate on the G7 for numerous reasons. They are an industrial country that requires a lot of energy for food production in factories and energy to meet household needs. As a result, clean energy plays a key part in their energy transition since it has various distinct qualities, such as abundant reserves, low emission levels, and the potential for widespread use.

The Driskrool Dray standard error estimator and panel quantile regression are used in this investigation. These methods outperform more traditional ones in estimating and extracting insights from estimations. Conventional instrumental variable techniques blatantly ignore the heterogeneity of the individual cross-sections, which evaluate the quantiles of the residuals using the median of the panel data as a whole. However, considering heterogeneity, the inference processes may yield more accurate insights. This problem is addressed by the quantile approach, which is thought to be better than commonly used estimating methodologies.

The study’s other sections are structured as follows: the first portion gives a general review of the trends in CO2 emissions and REN in the G7 nations, and the second section includes underlying literature based on previous studies. The following parts describe the methodology and estimates. The last section concludes and offers some advice to academics, researchers, and politicians.

## Theoretical underpinning and background

In today’s world, energy has transcended its role as a mere convenience to become an indispensable element of human survival. This elevated significance underscores the imperative to recognize energy access as a fundamental human right, paralleling other essential rights such as access to food, water, and healthcare. Just as individuals deserve justice in all aspects of life, they equally deserve access to modern energy sources, enabling them to lead dignified and fulfilling lives. This concept of energy as a right forms the cornerstone of the burgeoning energy justice movement.

At the heart of energy justice lies the equitable distribution of essential resources, encompassing the “primary goods” outlined by philosopher John Rawls. These primary goods encompass rights and liberties, powers and opportunities, and income and wealth (Rawls 1971, 1999). In his groundbreaking work, “A Theory of Justice,” Rawls proposes a distributive theory of justice grounded in the idea that rational individuals if placed behind a “veil of ignorance” that obscures their personal attributes and social standing, would opt for a system that maximizes the well-being of the least advantaged members of society. This principle, known as the “difference principle,” advocates for prioritizing the needs of those with the fewest resources, opportunities, and abilities (Rawls 1999).

In the context of energy, the energy justice movement strives to bridge the gap between those with and without access to modern energy sources, fostering a more equitable and just world. By recognizing energy access as a fundamental right, the movement seeks to ensure that all individuals, regardless of their socioeconomic status or geographical location, have the ability to utilize modern energy sources to meet their basic needs, pursue their aspirations, and fully participate in society (Sovacool and Dworkin 2015). The achievement of energy justice is not merely a matter of ensuring access to energy; it is about empowering individuals and communities to reap the transformative benefits of modern energy sources while safeguarding the environment for future generations. By recognizing energy access as a fundamental human right and pursuing a comprehensive approach to energy justice, we can create a more equitable and sustainable world for all.

However, yet, still, access to clean cooking technology, a form to transition to a low carbon economy, is a complex, frequently misunderstood subject that is frequently disregarded or oversimplified in world-scale decarbonization. Without concentrated attention, modeling studies might easily overlook its social intricacies, inherent interconnectedness with climate change, and varied character of the solutions(Dhingra et al. 2008; IRENA 2017). Overall, there is relatively little overlap between research that simulates energy sector decarbonization paths and studies that aim to provide universal clean cooking access. When both objectives are pursued concurrently, clean cooking availability is typically confined to a single technological solution, which is frequently not completely linked with the economy’s decarbonization pathway (Celik et al.; Ofori and Appiah-Opoku 2023). Furthermore, when modeling studies specifically target clean cooking targets, they often focus on evaluating the policy costs associated with the shift in a more conservative manner.

The fundamental goal of effective demand-side energy oversight is to alter long-standing patterns of energy use awareness and behavior (Ofori et al. 2023). To speed up the electrification and digitization of business, public transit, daily life, buildings, and other terminals, it is necessary to make advances in fundamental technologies like smart meters, utilities, and connectivity in advance (F. V. Bekun et al. 2023a, b; Ofori and Appiah-Opoku 2023). These innovations also call for creative market incentive mechanisms, like demand reaction and dynamic pricing.

Previous studies have noted that managing climate change, particularly in terms of CO_2_ reduction, necessitates reducing non-green (unclean) and increasing the quantities of green (clean) energy consumption, respectively (Bekun 2024; Gyamfi et al. 2022b; Radmehr et al. 2022; Yang et al. 2022a, b, c). However, to commence this compositional change in the energy mix, suitable technologies must be developed (Zhao et al. 2022) so that green energy can be generated and provided at lower prices than traditional non-green energy sources. As a result, investment in green energy development is postulated to aid in the restoration of environmental welfare (Alola et al. 2023a, b; Gyamfi et al. 2022a, b; Peng et al. 2022).

Beckerman coined the phrase “too poor, too green” (Beckerman 1992), suggesting that only affluent nations can use green technology to address environmental challenges and that impoverished countries lack the resources to safeguard the environment. Environmental technologies (ETs) must be developed and implemented to prevent future ecological deterioration, drastically cut emissions, and stop climate change. Innovation in ETs is crucial in industrializing nations like the G7 that are infamous for their unsustainable production methods but possess the economic heft needed to scale up ETs quickly. Multilateral climate change strategies continue to concentrate exclusively on technology transfer rather than cooperation and co-invention, even though the G7 currently contributes a significant portion of low-carbon technologies. As such, our research question seeks to inquire if clean energy and technology can address the environmental deficit in G7.

Additionally, the G7 nations have shown their commitment to investing in various clean energy projects and expressed a desire to work together internally to invent and disseminate ETs, indicating that internal cooperation may be an essential factor in the development of innovations. In light of this, we perform an empirical study of the G7 between 2000 and 2020 to show how technical cooperation among G7 nations considerably impacts the environment. We also give practical support for the need to promote more green technologies.

Also, the 2019 coronavirus (COVID-19) has substantially impacted the energy mix and temporarily lowered emissions of nitrogen dioxide and particulate matter (Anjum 2020; Zhang et al. 2021). For instance, during COVID-19, the proportion of clean energy used to generate power in member states of the European Union fell by 20% while the proportion of renewable energy sources rose by 9% (Strielkowski et al. 2021). However, the growing usage of clean energy came to the forefront during the energy crisis brought on by the hostilities between Russia and Ukraine (Yergin 2020). The International Energy Agency (IEA) has recommended boosting investment in clean power plant development to increase clean power output in its plans to alleviate the present energy crisis (Alola and Rahko 2024; Hoang et al. 2021). Given the effects of the current energy crisis and the IEA’s recommendations, clean power and renewable sources must be considered in future research on environmental quality. Additionally, clean energy does not utilize fossil fuels and retains clean elements when generating electricity. As a result, it does not emit GHGs, which is a significant benefit over fossil sources in terms of how it affects the quality of the environment (Tian et al. 2022).

## Methodology

The imperative drives the persistent focus on sustainable energy sources to curtail greenhouse gas emissions. Based on the assumptions stated above, we demonstrate empirically how clean energy and technology may be utilized to mitigate the negative consequences of CO_2_ emissions moderated for under-improved human development and the availability of technological innovation.

### Data source, model specification, and econometric methodology

#### Data source and variable description

The World Bank’s World Development Indicator website provided the data for this research, which covered the years 2000 through 2020, except for human development, which data is taken from UNDP.

**Fig. 1:**
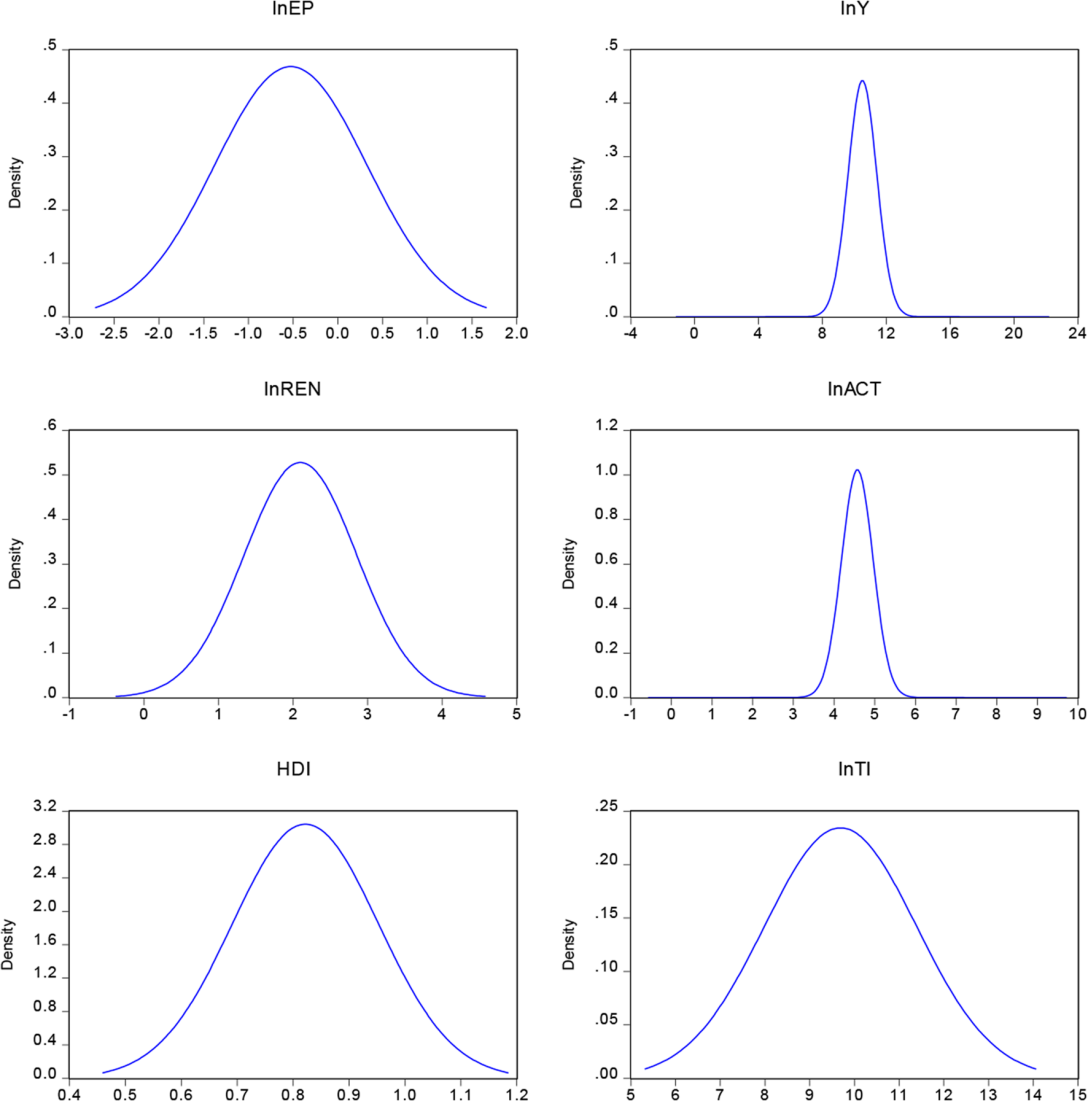
Graphical normality display of data

Environmental condition is quantified using CO_2_ emissions, which are estimated as CO_2_ emissions per capita in pursuit of the UN-SDG 13. This indicator serves as our dependent variable. The independent variables are renewable energy, a proxy for wind, hydro, and solar energy, and “access to clean energy and technology for cooking” (this component was added to address the data bias when selecting indicators for green energy). These advocate for the UN-SDG 7. Gross domestic product was used as a proxy for economic growth and was also in line with UN-SDG objective 8. Human development, a default for UN-SDG 3 and 4, served as the control variable. Technological innovation was also included as a control variable, which was proxied by patents. Table 1 displays an overview of the variables, symbols, definitions, measures, and data sources. Figure 1 also shows the distribution curve of each date.

**Table 1:** Description of data, sign, and source

Variable name	Sign	Description	Source
Environmental pollution	lnEP	CO_2_ emissions (metric tons per capita)	WDI
Economic growth	lnY	GDP per capital 2017	WDI
Renewable energy consumption	lnren	Renewable energy consumption % of final energy consumption	WDI
Access to clean energy	lnAct	The total population with access to clean fuels and technologies	WDI
Human development index	HCI	Human development index	UNDP
Technological innovation	lnTI	Patent of residents	WDI

### Conceptual model

Our models were in fivefolds, and the process adopted was a stepwise process for each model; we introduced additional variables to ascertain if it presented a new perspective to the earlier relationship. Also, this presented an opportunity to integrate policy initiatives that fit into the analytical process as a whole. This process enables better analysis of the hypothesis. The formulated equation below (see Eqs. (1) to (6)) provided the framework to test the hypothesis.

Theoretical analysis suggests that renewable energy may lessen the effects of economic expansion (Lu et al. 2022; Rehman et al. 2022), and human growth is the finest way to improve such a relationship (Hao 2022; Wen et al. 2022).

- Model 1: deciphering growth patterns in the G7.
  The inaugural model delves into the existing growth patterns prevalent in the G7, a group of leading industrialized nations. This analysis aims to identify the underlying trends and dynamics that characterize economic growth within these countries. By understanding the historical patterns of growth, we can establish a baseline from which to assess the potential impact of clean energy and green development strategies.
- Model 2: unraveling the conflict resolution potential of clean energy.
  The second model investigates whether the adoption of clean energy sources can mitigate, if not eliminate, the inherent conflict between economic growth and environmental sustainability. This analysis will examine the potential of clean energy to decouple economic growth from environmental degradation, thereby fostering a more harmonious relationship between these two critical domains.
- Model 3: exploring the nexus between renewable energy access and green development.
  The third model scrutinizes the potential of enhanced renewable energy access to stimulate green development and sustainable design practices. This analysis will assess whether the increased availability of renewable energy sources can catalyze the adoption of environmentally friendly technologies, processes, and infrastructure, leading to a more sustainable economic trajectory.
- Model 4: unmasking the influence of human growth on the overall interrelationships.
  The fourth model delves into the intricate relationship between human population growth and the dynamics of economic growth, clean energy adoption, and green development. This analysis will examine how population growth influences the demand for energy resources, the environmental impact of economic activities, and the overall pace of green development.

Multiple regression

1 $$Model:{Dep}_{it}={\beta }_{0}+{\beta }_{1}indep+\mu {Controls}_{it}+{\gamma }_{i}+{\sigma }_{it}+{\varepsilon }_{it}$$

2 $$Model\;1\;{EP}_{it}={\beta }_{0}+{\beta }_{1}{Y}_{it}+{\varepsilon }_{it}$$

3 $$Model\;2\;{\beta }_{0}+{\beta }_{1}{Y}_{it}+ {\beta }_{2}{REN}_{it}+{\varepsilon }_{it}$$

4 $$Model\;3.:{ES}_{it}{\beta }_{0}+{\beta }_{1}{Y}_{it} + {\beta }_{2}{REN}_{it}+{\beta }_{3}{ACT}_{it} +{\varepsilon }_{it}$$

5 $$Model\;4:{ES}_{it}= {\beta }_{0}+{\beta }_{1}{Y}_{it} + {\beta }_{2}{REN}_{it}+{\beta }_{3}{ACT}_{it} + {\beta }_{4}{HDI}_{it}+ +{\varepsilon }_{it}$$

6 $$Model\;5:{ES}_{it}= {\beta }_{0}+{\beta }_{1}{Y}_{it} + {\beta }_{2}{REN}_{it}+{\beta }_{3}{ACT}_{it} + {\beta }_{4}{HDI}_{it}+ {\beta }_{5}{TI}_{it}+{\varepsilon }_{it}$$

where *i* indexes a G7 states and *t* indexes are time period from 2000 to 2020.

The constant term is denoted by${\beta }_{0}$. The parameters to be estimated are denoted by ${\beta }_{1}$,${\beta }_{2}$…..${\beta }_{5}$. The error term is denoted by$\upvarepsilon$.

### Econometric methodology

The characteristics of the dataset and the econometric strategy used in this research are complementary. Before checking for the stationarity and cointegration features of the variables, we first looked into the potential of cross-sectional dependence (CD). The prediction of the elasticities using the proper regression techniques comes next. Finally, a causality analysis is carried out to see how it will affect policy.

### Cross-sectional dependency

We believe that because of their mutual economic interdependence and globalization, the influence of certain macro-economic variables on one nation may spread to the others. The G7 link may also encourage reliance including the cross-sectional units in the panel data set under consideration in this research. Consequently, according to Murshed et al. (2020), we first perform a CD test since the presence of CD might lead to inaccurate and misleading findings. The CD test suggested by Pesaran (2004) is used in this respect. The corresponding Pesaran (2004) CD test statistic is as follows.

### Unit root technique

The stationarity analysis is employed to determine how the factors are integrated into one another (Ma et al. 2022). Since using non-stationary data produces false results, it is important to determine the unit root qualities. The Im-Pesaran-Shin (IPS) test (Im et al. 2003) and other traditional first-generation stationarity tests might provide skewed results because of CD issues with the data. The second-generation stationarity tests were thus chosen to be used after Vural (2021). To provide reliable results, this research uses the CIPS and CADF techniques. Furthermore, any test accepts panels with either T > N or N > T. To calculate the CIPS test statistic, Pesaran (2007) proposed the equation shown below:

## Outcomes and discussion

### Descriptive statistics

In this section, the authors present the general overview of the distribution of the dataset used in this paper. The article investigates the environmental impact of some macro-environmental and economic indicators prior to the Cops 27 in G7 economies. To begin with, the data distribution using descriptive statistics is presented in Table 2. The log was applied to all the variables to allow for the estimated coefficients to be interpreted as elasticities. Each factor has a higher degree of variation. The skewness test outcomes reveal that all the coefficients are skewed to the left. Consequently, the correlation analysis was done to investigate the multicollinearity intensity (Table 3). Indeed, the results reveal that economic growth, access to clean energy, and human capital index correlate negatively with environmental pollution, while renewable energy and technological innovation have a positive interaction with ecological pollution.

**Table 2:** Descriptive statistics

Variables	Obrs	Mean	Stad. dev	Minim	Maxim	Skewen	Kurte
lnEP	147	− 0.525	0.852	− 2.164	0.695	− 0.451	1.646
lny	147	10.503	0.902	− 0.148	11.013	− 11.315	134.293
lnren	140	2.098	0.756	− 0.163	3.122	− 0.958	4.022
lnAct	147	4.573	0.389	− 0.117	4.605	− 12	145.007
hci	147	0.822	0.131	0.491	0.948	− 1.116	2.739
lnTi	140	9.689	1.702	6.661	12.726	− 0.059	2.141

**Table 3:** Correlation matrix

	lnEP	lny	lnren	lncook	hci	lnpnr
lnEP	1					
lny	− 0.0607	1				
lnren	0.489^***^	0.246^**^	1			
lnAct	− 0.0771	0.983^***^	0.252^**^	1		
hci	− 0.509^***^	− 0.0626	0.00345	− 0.0405	1	
lnTi	0.00481	0.0727	− 0.143	− 0.0404	− 0.071	1

### Cross-sectional dependency tests

Subsequent to the correlation analysis estimation, we perform the CD test to ascertain the spillover effect in the data (Table 4). It is imperative to note that a cross-sectional dependence indicates that a marginal variation in any of the coefficients of one country is likely to result in a corresponding effect on that of other countries. From Table 4, it is revealed that all the variables are statistically significant at 1 percent for all the tests except technological innovation, that is insignificant under the Pesaran CD test. We can, therefore, conclude in favor of the spillover effect in the data. Next, we executed the unit root test (Table 5). To achieve this, the CIPS and CADF unit root tests were employed. The result reveals that except for access to clean energy and human capital index, which are both I(0) and I(1), all other coefficients are I(1) under the CIPS. On the contrary, the result under the CADF suggests that with the exception of ecological pollution and access to clean energy, all other coefficients are I(0) and I(1).

**Table 4:** Cross-sectional dependency

Variable	Breusch-Pagan LM	Pesaran scaled LM	Bias-corrected scaled LM	Pesaran CD
lnEP	290.010***	41.509***	41.334***	16.890***
lny	223.868***	31.303***	31.128***	8.975***
lnren	271.302***	38.622***	38.438***	15.468***
lnAct	315.000***	45.365***	45.190***	15.000***
hci	410.202***	60.055***	59.880***	20.241***
lnTi	153.307***	20.415***	20.240***	− 0.817

**Table 5:** Panel unit root test

	CIPS(0)	CIPS(1)	CADF(0)	CADF(1)
lnEP	− 2.089	− 3.995***	− 0.236	− 6.838***
lny	− 2.089	− 6.175***	− 5.077***	− 11.143***
lnren	− 3.39	− 5.011***	− 2.923**	− 8.927***
lnAct	2.61***	2.61***	11.014	9.743
hci	− 2.575**	− 3.259***	− 1.977*	− 5.167***
lnTi	− 1.502	− 4.083	− 1.750*	− 7.922***

Finally, the cointegration and the slope homogeneity test (see Table 6 and 7) were investigated. The cointegration technique was employed to ascertain the long-run effect regarding the coefficients. The Kao residual cointegration test was applied to test the long-run linkage (Table 6), and the Westerlund–Homogeneity slope test was employed to assess the slope homogeneity.

**Table 6:** Kao residual integration test

	t-Statistic	Prob
ADF	− 1.77268	0.038**
RESID(− 1)	− 3.88315	0.000***
D(RESID(− 1))	2.939445	0.004**

**Table 7:** Westerlund–homogeneity slope test

	Delta	*p*-value
	4.996***	0.000
Adj	6.010 ***	0.000

### An overview of the empirical results

Following the outcomes from the preliminary assessments, we proceed with the estimation of the study’s main results. As stated before, two estimation procedures, namely, the results of Driscoll-Kraay standard errors with fixed and random effects and the panel quantile estimators, were applied to address the study’s main objective. The outcomes from the Driscoll-Kraay estimator are presented in Table 8. As shown in Table 8, five (5) models were estimated for the Driscoll-Kraay standard errors with fixed effect, while one (1) model was estimated for the random effects of the robustness check.

**Table 8:** Results of models 1–4 using Driscoll-Kraay standard errors (fixed and random)

	Driscoll-Kraay fixed effects					Random effects
	Model 1	Model 2	Model 3	Model 4	Model 5	Model 6
lny	− 0.0221**	0.0433***	− 1.3066***	− 1.2304***	− 1.1233***	− 1.1176***
	(− 2.10)	(− 6.43)	(− 7.46)	(− 7.42)	(− 4.70)	(− 5.02)
lnren		− 0.2025***	− 0.1198***	− 0./50746***	− 0.0897***	− 0.0879***
		(− 18.45)	(− 8.40)	(− 5.99)	(− 4.53)	(− 6.38)
lnAct			3.0321***	2.8340***	2.5969***	2.5830***
			− 7.88	− 7.78	− 4.93	− 5.22
hci				− 1.2560***	− 1.0007***	− 1.0555***
				(− 5.25)	(− 3.20)	(− 3.30)
lnTi					− 0.1144**	− 0.1103**
					(− 2.22)	(− 2.20)
Constant	− 0.2933***	− 0.5454***	− 0.4070***	0.6341***	1.5066***	1.5143**
	(− 3.73)	(− 8.32)	(− 6.85)	− 3.28	− 4.06	− 2.49
No. of observations	147	140	140	140	133	133
*R*-squared						
*F* statistic	4.408	240.797	347.309	728.676	780.405	

For model 1, econometric growth showed a propensity to lessen carbon emissions. This is an indication of green growth, which is encouraging in the G7 states, and the policy which led to such a phenomenon is well intended and should be encouraged.

In model 2, when renewable energy was introduced, it showed that economic growth leads to an expansion in environmental pollution, but renewable energy reduces carbon emissions. These results, on the plain surface, are regular but also show a factor that gives credence to the need to encourage green growth, which denotes increasing the energy mix of production with more renewable sources.

In model 3, here, access to clean energy was accounted for in the equation. The finding showed a propensity for green growth as it reduces carbon emission; renewable energy also leads to improved environmental quality, but LnAct leads to an increase in carbon emission. This could be attributed to poor policy implementation, leading to no yield at the moment. Hence, it is imperative to encourage policymakers to continue providing clean energy and technology to households and businesses. As the literature indicates, such components improve the environment in the long run.

In model 4, here, the moderating role of human development was introduced. Again, economic growth showed a propensity to reduce carbon emission; renewable energy also helps reduce carbon emission, lnact added to the bane of the environment, and human development also helped minimize environmental pollution. This is evidenced in the literature as an educated populace turns to gravitate toward more sustainable consumption and production, which serve the good of society.

In model 5, technological innovation was introduced to the equation. All variables showed a propensity to reduce environmental pollution except for clean energy and technology access. This could be due to policy failure calling for G7 states to go back to the drawing board and develop sound strategies in the deployment of clean energy to supplement goals of achieving the UN-SDG 13 on the back of Un-SDG 7.

The statistical finding indications are detailed as follows.

The outcomes show that economic growth utilizes a significant negative influence on ecological pollution at a 1% level of significance under all the models except in model 2, where a positive effect is recorded. This implies that for every percentage increase in economic growth, a corresponding increase in ecological pollution is achieved between the range of 0.02% and 1.31%. Also, a cursory inspection of the result shows that model 3 expresses the largest elasticity of economic growth, with the lowest being that in model 1, where only economic growth is considered. Similarly, renewable energy is documented to interact negatively with ecological pollution (see models 3 to 6). This suggests that increasing renewable energy consumption in G7 economies will considerably reduce ecological pollution by a margin ranging between 0.07 and 0.20%. It is imperative to note that model 2 produces the largest elasticity of renewable energy consumption, whereas model 4 generates the lowest elasticity.

Furthermore, the human capital index has a significant negative coefficient under models 4 to 6, suggesting that it mitigates ecological pollution in G7 economies. This suggests that every 1% growth in the human development index would likely correspond to a significant decrease in ecological pollution with a 1 to 1.26% percentage range. Additionally, technology innovation is reported to have a negative and significant bearing on ecological pollution. Thus, a percentage growth in technological innovation is likely to have about a 0.11% increase in ecological pollution in G7 countries. On the contrary, access to clean energy and technology has a significant positive impact on ecological pollution. This result implies that as G7 countries increase access to clean energy and technology, ecological pollution surprisingly grows by a range between 2.58 and 3.02%. In other words, access to clean energy and technology aggravates ecological pollution.

Subsequently, the panel quantile regression analysis was performed to investigate the environmental impact of the study variables, given that ecological pollution levels vary from one country to another, which may imply the application of different environmental policies at varying degrees to achieve improved environmental quality. For this reason, Table 9 displays the result of the panel quantile regression. To achieve this goal, nine (9) quantiles ranging from 0.1 to 0.9 were classified under three main groups: lower, median, and upper. Thus, quantiles 0.1 to 0.4, 0.5, and 0.6 to 0.9 are classified as the lower, median, and upper quantiles, respectively. In other words, the lower quantile implies countries with lower levels of ecological pollution, while the upper quantile signifies countries with higher levels of ecological pollution. The result shows that economic growth only exerts a significant negative impact on ecological pollution in the first quantile but positively promotes pollution in the median and upper quantile groups. The result suggests that countries with lower levels of environmental pollution are likely to generate positive environmental externalities from increases in economic growth. In comparison, those with higher pollution levels generate negative environmental externalities. Contrary to the result of access to clean energy and technology (Table 8), Table 9 shows a decreasing effect under the median and upper quantiles. Interestingly, access to clean energy and technology generates greater ecological externalities under the upper quantiles (3.42% and 3.54% under the 6th and 9th quantiles, respectively) than under the median quantile (2.77%). Furthermore, the human capital index was found to exert a significant negative impact across all the quantiles. However, a cursory inspection of the results reveals that higher environmental externalities are reaped from the lower quantile (0.1 to 0.3) compared with the median and upper quantiles. In contrast, surprisingly, the findings on renewable energy encourage ecological pollution under all the quantiles, with the higher impact recorded in the upper-lower quantile (0.4), median (0.5), and all the upper quantiles. Finally, the result reveals that while technological innovation exerts a significant positive effect on ecological pollution under the lower quantiles, it may significantly and negatively impact it under the upper quantiles.

**Table 9:** Results from the panel quantile regression model

	Lower quantile				Median	Upper quantile			
	Q.0.1	Q.0.2	Q.0.3	Q.0.4	Q.0.5	Q.0.6	Q.0.7	Q.0.8	Q.09
lny	− 0.706**	− 0.381	− 0.084	0.387	1.015*	1.364***	1.308***	0.595	− 0.05
	(− 2.40)	(− 1.12)	(− 0.17)	− 0.62	− 1.75	− 2.85	− 2.77	− 1.43	(− 0.19)
lnren	0.415***	0.357***	0.421***	0.540***	0.560***	0.613***	0.657***	0.690***	0.687***
	− 7.69	− 5.72	− 4.57	− 4.71	− 5.25	− 6.98	− 7.59	− 9.03	− 14.62
lnAct	1.063	0.379	− 0.297	− 1.373	− 2.765**	− 3.544***	− 3.423***	− 1.809*	− 0.327
	− 1.57	− 0.49	(− 0.26)	(− 0.96)	(− 2.07)	(− 3.22)	(− 3.16)	(− 1.89)	(− 0.56)
hci	− 6.713***	− 6.290***	− 5.706***	− 4.277***	− 2.934***	− 2.183***	− 2.630***	− 4.307***	− 5.736***
	(− 19.62)	(− 15.92)	(− 9.77)	(− 5.89)	(− 4.34)	(− 3.92)	(− 4.79)	(− 8.88)	(− 19.24)
lnTi	0.212***	0.158***	0.132**	0.062	− 0.021	− 0.07	− 0.103**	− 0.168***	− 0.183***
	− 7.03	− 4.53	− 2.56	− 0.97	(− 0.36)	(− 1.41)	(− 2.13)	(− 3.93)	(− 6.97)
Constant	4.009***	4.162***	3.886***	3.308**	2.917**	2.719***	3.485***	5.742***	7.251***
	− 6.52	− 5.86	− 3.7	− 2.54	− 2.4	− 2.72	− 3.53	− 6.59	− 13.54

## Results and discussion

In this section, we present an insight into the findings of the study. The study reveals that ecological pollution is greatly influenced by economic growth. However, contrary to existing studies, economic growth in this study generally mitigates ecological pollution. This could be explained using the analogy of the environmental Kuznets curve, which posits that ecological pollution declines beyond a given threshold of development. In the same vein, it is plausible that G7 economies, which are largely classified as developed economies, are at the stage of economic development where any marginal increase in economic growth is likely to result in a corresponding decrease in pollution. This result is in line with the findings of Ozturk et al. (2022) but contradicts the findings of the literature (Ali and Amfo 2021; Ali and Anufriev 2020; Ali et al. 2022b; Huang et al. 2021a, 2021b; Radmehr et al. 2022). However, in another vein, the result suggests that with the exception of the first quantile, economic growth largely inhibits environmental quality at different levels of ecological pollution, as shown in Table 9. This result could be attributed to the various quantiles representing the early stages of economic development, where any marginal increase in growth would lead to environmental degradation.

The role of renewable energy consumption in pollution mitigation has been extensively discussed in the literature. Renewable energy is believed to be one of the sure ways to achieve sustainable economic growth without jeopardizing environmental quality. Indeed, our results support the findings of the literature (Ali et al. 2022b; Ali et al. 2022a; Haldar and Sethi 2022; Raihan and Tuspekova 2022; Sahoo and Sethi 2022; Haldar and Sethi (2021);;; who argue that increases in the consumption of renewable energy is likely to correspond to decreases in pollution levels. With regard to the human capital index, we observe a boost in environmental quality via a reduction in ecological pollution levels. Thus, a 1% rise in the human capital index will mitigate ecological pollution by 1.25%, 1.00%, and 1.05% for models 3 to 5. This concords with Radmehr et al. (2022) for G7 countries, Ahmed and Wang (2019) for India, Ahmed et al. (2020) for China, Huang et al. (2021b, a) for emerging economies, Chen et al. (2019) for CEE countries, Tiwari et al. (2022) for China and Brazil, and that of the results in the panel quantile regression (Table 9). Finally, the finding on access to clean energy and technology implies a harmful effect on the environment which contradicts our priori expectation in Table 8 but confirms the same in Table 9. The result suggests that access to clean energy and technology should not be viewed from an economic bloc perspective but from the point of view of emission levels, as shown in Table 9. In other words, policies that seek to advance access to clean energy and technology must be implemented in relation to a country’s emission levels to achieve the desired results.

## Conclusion

Clean energy, such as wind and solar power, produces little or no pollutants and can help reduce the chaotic issues of climate change. Additionally, it has the potential to generate employment prospects and economic possibilities. It can also create new jobs and economic opportunities, particularly in the renewable energy sector. In addition, clean energy technologies, such as energy-efficient appliances and buildings, can help reduce energy consumption and lower energy bills for households and businesses; as such, it is an important component when policymakers are seeking to improve the environment. This goal has also been one of the imperative discussions during all the cops meeting. Hence our study goal was to test how the prospect of clean energy and technology helps to address the bane of environmental degradation while being moderated under the conditions of human development and technological innovations.

Our results give credence to the idea that, in G7 economies, clean energy may aid in achieving the SDGs. By providing an empirical method, this article adds to the body of literature by demonstrating not only the ability of renewable energy to achieve these goals but also how it can be used to lessen the adverse effects of the four indicators of environmental degradation on economic growth and human development. This method also examines the consequences for practice and public policy and offers some recommendations for future study. In conclusion, clean energy and advanced technology have the capacity to greatly contribute to enhancing environmental sustainability within the G7 nations while concurrently fostering human progress. By transitioning to clean energy sources and implementing technology-based solutions, G7 countries can reduce their greenhouse gas emissions, conserve natural resources, and encourage economic growth.

As a policy recommendation, the G7 countries should prioritize the deployment of clean energy and technology-based solutions. This can be achieved through a combination of government funding and incentives, private sector investment, and international collaboration. By taking these steps, the G7 countries can promote environmental sustainability and human development.

It is also important for the G7 countries to work together and share their knowledge and expertise in clean energy and technology. By collaborating and sharing best practices, the G7 countries can accelerate the transition to clean energy and help to address global challenges such as climate change and resource scarcity.

Additionally, the G7 countries must engage with other stakeholders, including civil society organizations, businesses, and academia, in order to ensure that the transition to clean energy and technology-based solutions is inclusive and equitable. This will help to ensure that all members of society share the benefits of clean energy and technology and that no one is left behind.

In conclusion, the suggested policy guidelines to advance the adoption of clean energy and technology for addressing environmental sustainability in the G7 nations encompass the following.

I. Increase government funding and incentives for developing and deploying clean energy technologies.
II. Encourage private sector investment in clean energy and technology-based solutions through tax incentives and other financial incentives.
III. Facilitate international collaboration on clean energy and technology-related research and development.
IV. Engage with other stakeholders, including civil society organizations, businesses, and academia, to ensure an inclusive and equitable transition to clean energy and technology.
V. Develop and implement regulations and standards to support the transition to clean energy and technology-based solutions.
VI. Invest in education and training programs to build workforce expertise to support the clean energy and technology sectors.
VII. Promote the use of clean energy and technology-based solutions through public awareness and outreach campaigns.
VIII. Collaborate with other governments and international organizations to support the global transition to clean energy.
IX. Develop a plan to phase out the use of fossil fuels and transition to renewable energy sources over time.
X. Provide support and resources to help households and businesses make the transition to clean energy technologies.
XI. Monitor and evaluate the progress of clean energy initiatives, and adjust policies as needed to support their success.

By taking action at the national and individual levels and collaborating with other countries and stakeholders, the G7 countries can lead the way in promoting a sustainable and prosperous future for all.

## Figures and Tables

**Figure:**
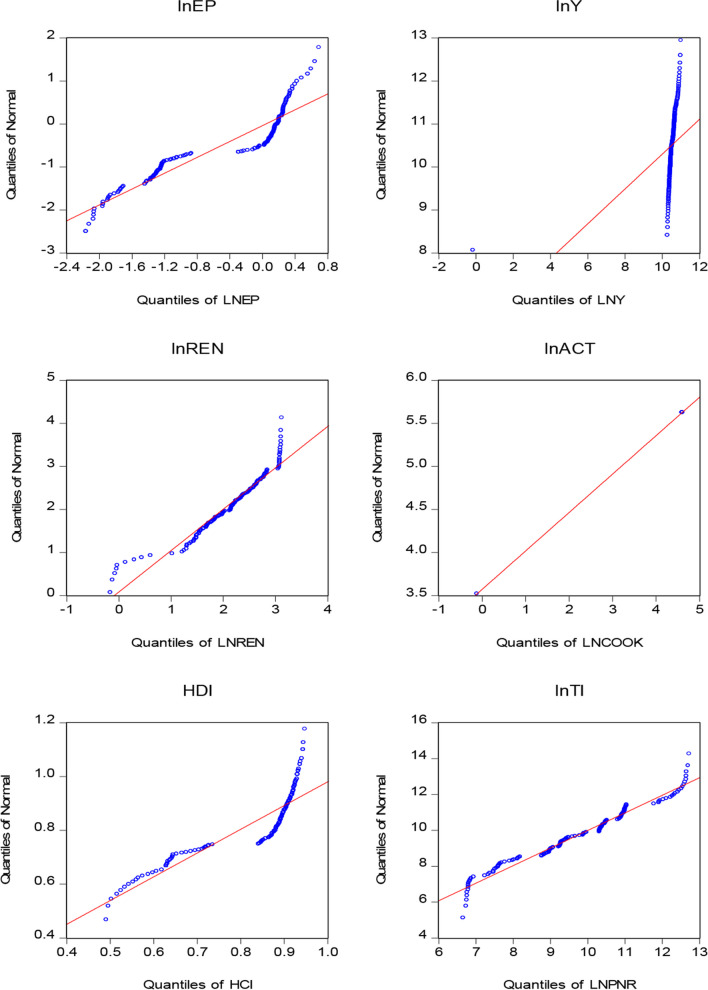
Distribution of Variable on interest

## Data Availability

The data are available upon demand by request to the corresponding author.
