# Correction to: Can clean energy and technology address environmental sustainability in G7 under the pre‑set of human development?

**DOI:** 10.1007/s11356-024-32266-5

**Published:** 2024-02-05

**Authors:** Shaibu Ali, Khatib Ahmad Khan, Bright Akwasi Gyamfi, Elvis Kwame Ofori, Derrick Tetteh, Zilola Shamansurova

**Affiliations:** 1 School of Finance and Economics, Jiangsu University, Xuefu Road, Zhenjiang, Jiangsu China; 2 School of Commerce and Management Studies, SunRise University, Alwar, Rajasthan India; 3 School of Business, Xi’an International University, Xi’an, China; 4 School of Management, Sir Pandampat Singhanian University, Udaipur, Rajasthan India; 5 Plants & Agribioscience, School of Biological & Chemical Sciences, Ryan Institute, University of Galway, Galway, Ireland; 6 School of Management Engineering, Zhengzhou University, Henan Province, Zhengzhou, People’s Republic of China; 7 School of Business, University of Cape Coast, Cape Coast, Ghana; 8 Department of Finance, Tashkent State University of Economics, Tashkent, Uzbekistan

**Correction to: Environmental Science and Pollution Research**

10.1007/s11356-024-32011-y

Upon thorough review, it has come to our attention that there was a mistake in the author's contribution details provided for the paper. The correct author contribution details should be as follows:

Shaibu Ali, PhD (conceptualization, data curation, methodology, formal analysis, writing-original draft)

Khatib Ahmad Khan (Methodology, formal analysis)

Bright Akwasi Gyamfi (visualization, data curation)

Elvis Kwame Ofori, PhD Candidate (formal analysis, writing-original draft)

Derrick Tetteh (Methodology, data curation)

Zilola Shamansurova (Methodology, data curation)

I sincerely apologize for any confusion or inconvenience this oversight may have caused. It appears that there was a mix-up in the submission process, particularly in the author contribution section. We are earnestly seeking your indulgence to make the necessary corrections in the records of the submitted papers.

